# Statin Use and Mortality among Patients Hospitalized with Sepsis: A Retrospective Cohort Study within Southern California, 2008–2018

**DOI:** 10.1155/2022/7127531

**Published:** 2022-05-06

**Authors:** Brannen Liang, Su-jau T. Yang, Kenneth K. Wei, Albert S. Yu, Brendan J. Kim, Michael K. Gould, John J. Sim

**Affiliations:** ^1^Department of Internal Medicine, Kaiser Permanente Los Angeles Medical Center, Los Angeles, CA, USA; ^2^Department of Research & Evaluation, Kaiser Permanente Southern California, Pasadena, CA, USA; ^3^Division of Pulmonary Critical Care Medicine, Kaiser Permanente Los Angeles Medical Center, Los Angeles, CA, USA; ^4^Department of Health Systems and Clinical Science, Kaiser Permanente Bernard J. Tyson School of Medicine, Los Angeles, CA, USA; ^5^Division of Nephrology and Hypertension, Kaiser Permanente Los Angeles Medical Center, Los Angeles, CA, USA

## Abstract

**Background:**

Despite early goal-directed therapy, sepsis mortality remains high. Statins exhibit pleiotropic effects.

**Objective:**

We sought to compare mortality outcomes among statin users versus nonusers who were hospitalized with sepsis.

**Methods:**

Retrospective cohort study of patients (age ≥18 years) during 1/1/2008–9/30/2018. Mortality was compared between statin users and nonusers and within statin users (hydrophilic versus lipophilic, fungal versus synthetic derivation, and individual statins head-to-head). Multivariable Cox regression models were used to estimate hazard ratios (HR) for 30-day and 90-day mortality. Inverse probability treatment weighting (IPTW) analysis was performed to account for indication bias.

**Results:**

Among 128,161 sepsis patients, 34,088 (26.6%) were prescribed statin drugs prior to admission. Statin users compared to nonusers had a 30-day and 90-day mortality HR (95% CI) of 0.80 (0.77–0.83) and 0.79 (0.77–0.81), respectively. Synthetic derived statin users compared to fungal derived users had a 30- and 90-day mortality HR (95% CI) of 0.86 (0.81–0.91) and 0.85 (0.81–0.89), respectively. Hydrophilic statin users compared to lipophilic users had a 30-day and 90-day mortality HR (95% CI) of 0.90 (0.81–1.01) and 0.86 (0.78–0.94), respectively. Compared to simvastatin, 30-day mortality HRs (95% CI) were 0.85 (0.66–1.10), 0.87 (0.82–0.92), 0.87 (0.76–0.98), and 1.22 (1.10–1.36) for rosuvastatin, atorvastatin, pravastatin, and lovastatin, respectively.

**Conclusion:**

Statin use was associated with lower mortality in patients hospitalized with sepsis. Hydrophilic and synthetic statins were associated with better outcomes than lipophilic and fungal-based preparations.

## 1. Introduction

Sepsis affects 1.7 million adults in the United States (US) annually contributing to greater than 250,00 deaths [1]. Total hospital costs for sepsis rose from $15.4 billion to $24.3 billion between 2003 and 2007 in the US [2]. Sepsis is defined as acute, life-threatening end-organ damage caused by a dysregulated immunologic response to infection [3]. Management strategies for sepsis include early fluid resuscitation, identification of infectious sources, measurement of biomarkers, administration of antibiotics, and the addition of hemodynamic support medications as necessary [4]. Despite early goal-directed therapy, the mortality rate among patients with sepsis is estimated to be as high as 28%, nationally [5].

Statins are recommended for primary and secondary prevention of cardiovascular events in patients with atherosclerotic cardiovascular disease or risk [6, 7]. They are also recommended as secondary prevention of stroke in patients with a history of ischemic stroke and dyslipidemia [8]. In 2013 alone, it is estimated that 39.2 million Americans were taking a statin, a 79.8% increase from 2003 [9]. Determining a potential statin benefit in sepsis patients within a real-world clinical environment inclusive of a large diverse population would add important insights and perhaps provide translational value in terms of clinical decision-making [10].

Statins have exhibited pleiotropic effects including potential benefit in sepsis. Previous observations have suggested that statins may attenuate the severity of sepsis [11, 12]. In addition, anti-inflammatory, immunomodulatory, and anticancer effects have been attributed to statins [13]. Antimicrobial properties of statins has been demonstrated in vitro and in animal models [13]. However, the precise mechanisms of statins' role in sepsis remain unclear. Nevertheless, both observational and interventional trials have studied statin use for sepsis outcomes [11, 12, 14, 15]. Many of these studies have concentrated on sicker patients, homogeneous populations, and focused on a specific statin class. Whether specific statin classes affect sepsis outcomes differently may also provide insights into mechanisms and potential management strategies.

We leveraged the large race/ethnically diverse population of an integrated health system with comprehensive electronic health records inclusive of pharmacy records to undertake this study. We hypothesized that patients hospitalized with sepsis and were prescribed statins prior to admission would have improved outcomes compared to nonstatin users. Thus, we compared mortality outcomes among patients hospitalized with sepsis who were statin users and nonstatin users. Additionally, we compared outcomes among users of specific statins and statin class/properties in sepsis.

## 2. Methods

### 2.1. Data Source

We performed a retrospective cohort study within Kaiser Permanente Southern California (KPSC), which is an integrated health system that provides care to more than 4.7 million members at 15 medical centers and greater than 200 medical offices, spanning across 10 counties of Southern California [16]. KPSC is a prepaid integrated healthcare plan, and members have equal access to healthcare services, benefits, clinic visits, and medications. The KPSC electronic health records capture information from routine clinical practice, including demographics, comorbidities, and utilization. The KPSC member population is sex/gender balanced and is also ethnically, racially, and socioeconomically diverse, representative of the Southern California population from which it is drawn [16]. Medications were identified using generic product identifier codes from the KPSC pharmacy analytic database, which provides information on medications prescribed and dispensed. Over 95% of members obtain their medications from KPSC pharmacies. This study received approval by the Institutional Review Board of KPSC (#12145). Informed consent was waived for this deidentified electronic research database.

### 2.2. Study Cohort

**Figure 1:**
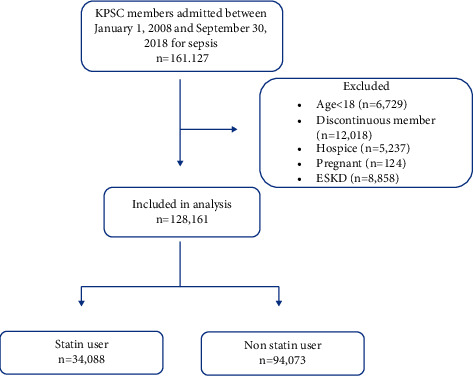
Study cohort. 128,161 KPSC members admitted to the hospital with sepsis from January 1, 2008, to September 20, 2018, were included in the analysis after the appropriate exclusion criteria were applied. Of this population, 34,088 patients were taking statins and 94,073 patients were not taking statins.

Individuals ≥18 years with continuous KPSC membership for ≥180 days were included in the study if they were admitted to the hospital with a diagnosis of sepsis (identified based on explicit ICD-9-CM and ICD-10-CM codes) within the KPSC healthcare system from January 1, 2008, to September 30, 2018 (Figure 1, Supplemental Table 1). The date of admission for sepsis was considered the index date. Those designated as hospice patients, individuals with end stage kidney disease, and pregnant women were excluded from the study. Recurrent admissions by the same patient for a diagnosis of sepsis were also excluded.

### 2.3. Medication Exposure

The KPSC pharmacy analytic database was used to evaluate medication dispensation. Statin use was defined by fill of at least two prescriptions for a statin within six months prior to admission, with a second prescription filled within 30 days of admission. Seven statin preparations were included in the study: atorvastatin, fluvastatin, lovastatin, pitavastatin, pravastatin, rosuvastatin, and simvastatin. Statins were further categorized by their derivation and biochemical properties (Table 1).

### 2.4. Outcomes

The primary outcome was defined as 30-day all-cause mortality. The secondary outcome was 90-day all-cause mortality. The secondary outcome was length of hospital stay.

### 2.5. Analysis

Primary and secondary outcomes were assessed among statin users and nonusers. Further analysis was then performed among all statin users. Outcomes were assessed among individual statins head-to-head (with simvastatin as reference) and by derivation (fungal or synthetic) and biochemical property (hydrophilic or lipophilic). Patients were followed from the index date to occurrence of death and/or the end of the study period. For bivariate analysis, we used Pearson' chi-square test for categorical variables and the Wilcoxon rank-sum test for continuous variables. Cox regression models adjusting for covariates were used to estimate hazard ratios (HR) for mortality. Adjusted variables included age, sex, race/ethnicity, liver disease, chronic kidney disease, heart failure, ischemic heart disease, chronic pulmonary disease, rheumatological disease, hypertension, cerebrovascular disease, diabetes, dementia, malnutrition, peptic ulcer disease, any malignancy, organ transplantation, history of gastrointestinal hemorrhage, alcohol/drug use, HIV/AIDS, and hyperlipidemia. Specific medications adjusted for included biologics, systemic corticosteroids, and disease modifying antirheumatologic drugs (Supplementary Table 2). Any missing data were not included in the analyses. There were not imputations performed in the data analyses.

We performed an inverse probability treatment weighted (IPTW) propensity score analysis to account for indication bias and in an attempt to mimic a randomized intervention approach. Standard errors within IPTW modeling were estimated using the sandwiching method. Determination of balance was attained as the absolute weighted standardized difference was equal to or less than 0.10 for all covariates. All statistical analyses were performed using SAS version 9.4 (SAS Institute, Cary, NC, USA), and a *p* value less than 0.05 was considered statistically significant.

## 3. Results

### 3.1. Study Population

A total of 128,161 patients admitted to the hospital with a diagnosis of sepsis were identified for the study (Table 2). The mean age of the cohort was 66.9 (±17.9) years with 51% of the population being female. Whites, Blacks, Hispanics, and Asian/Pacific Islanders comprised of 51%, 11.8%, 27.9%, and 7.9% of the cohort, respectively. The most common comorbidities were hypertension (71.5%), hyperlipidemia (64.8%), diabetes (41.2%), malnutrition (34.6%), chronic kidney disease (31.2%), and ischemic heart disease (26.4%). The most common nonstatin classes of medications were beta-blockers (37.3%), ACE inhibitors (34%), and angiotensin II antagonists (12.6%).

### 3.2. Statin vs. No Statin Population

Among the study population, 34,088 (26.6%) patients received statin therapy and 94,073 (73.4%) did not receive statin therapy prior to the index admission for sepsis. The statin user population was older, male predominant, and included more Whites. Overall, Charlson comorbidity index (CCI) scores were higher in statin users compared to nonstatin users, where statin users had CCI score of ≥4 in 53.4 compared to 33.7% in nonstatin users (Table 2). There were differences in comorbidities such as heart failure, ischemic heart disease, hypertension, cerebrovascular disease, and diabetes. Beta-blocker and renin angiotensin system inhibitor use also significantly differed.

### 3.3. Outcomes

#### 3.3.1. Study Population (Statin and Nonstatin Users)

A total of 19,173 (15%) patients died within 30 days and 26,226 (20.4%) patients died within 90 days. Mean length of hospital stay for the entire population was 7 days, SD (10.5). Crude mortality rates among statin users were 5,032 (14.8%) within 30 days and 6,951 (20.4%) within 90 days. Mean length of hospital stay was 6.6 days, SD (8.9). Among nonstatin users, the crude mortality rates were 14,141 (15%) within 30 days and 19,275 (20.5%) within 90 days. Mean length of hospital stay was 7.2 days, SD [11].

After multivariable adjustment for age, sex, race/ethnicity, liver disease, chronic kidney disease, heart failure, ischemic heart disease, chronic pulmonary disease, rheumatological disease, hypertension, cerebrovascular disease, diabetes, dementia, malnutrition, peptic ulcer disease, any malignancy, organ transplantation, history of gastrointestinal hemorrhage, alcohol/drug use, HIV/AIDS, and hyperlipidemia, specific medications adjusted for included biologics, systemic corticosteroids, and disease modifying antirheumatologic drugs, the mortality HRs (95% CI) were 0.80 (0.77–0.83) and 0.79 (0.77–0.81) for statin users compared to nonusers at 30-day and 90-day, respectively (Table 3).

Among statin users, the adjusted HRs (95% CI) were 0.86 (0.81–0.91) and 0.85 (0.81–0.89) for synthetic-derived statin users compared to fungal-derived statin users at 30-day and 90-day, respectively. For hydrophilic statin users compared to lipophilic statin users, the adjusted HR's (95% CI) were 0.90 (0.81–1.01) and 0.86 (0.78–0.94) for 30-day and 90-day mortality, respectively (Table 3, Supplementary Table 3).

Comparison of individual statins was performed using simvastatin as reference. The adjusted HRs (95% CI) for mortality at 30 days were 0.85 (0.66–1.10), 0.87 (0.82–0.92), 0.87 (0.76–0.98), and 1.22 (1.10–1.36) for rosuvastatin, atorvastatin, pravastatin, and lovastatin, respectively, compared to simvastatin. The adjusted HRs (95% CI) for mortality at 90 days were 0.77 (0.62–0.97), 0.83 (0.74–0.92), 0.85 (0.81–0.90), and 1.19 (1.08–1.31) for rosuvastatin, pravastatin, atorvastatin, and lovastatin, respectively, compared to simvastatin.

### 3.4. IPTW Analysis

**Figure 2:**
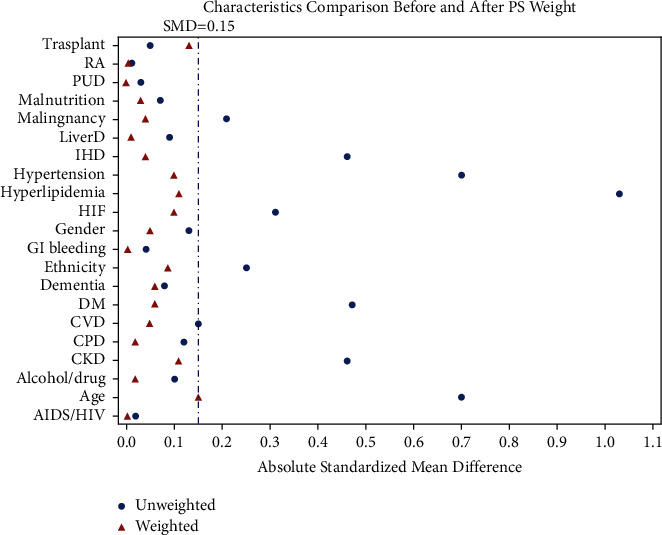
Comparison of weighted and unweighted covariates after IPTW analysis. Covariates adjusted for in the IPTW analysis are listed on the *y*-axis. ^∗^RA, rheumatological disease; PUD, peptic ulcer disease; LiverD, liver disease; IHD, ischemic heart disease; HF, heart failure; GI, gastrointestinal; DM, diabetes mellitus; CVD, cardiovascular disease; CPD, chronic pulmonary disease; CKD, chronic kidney disease; AIDS/HIV, acquired immunodeficiency syndrome/human immunodeficiency virus.

In the IPTW analysis, the hazard of death was 13% lower in statin users compared to nonusers at 30 days (HR 0.87, 95% CI 0.84–0.89) and at 90 days (HR 0.87, 95% CI 0.85–0.90). Comparison of weighted and unweighted covariates adjusted for the IPTW analysis is shown in Figure 2.

## 4. Discussion

Within a large, diverse population of a real-world clinical environment, we evaluated the relationship between statin use and mortality among 128,161 patients admitted to the hospital with sepsis. Among this population, statin users were observed to have 20% lower 30-day and 90-day mortality when compared to nonusers. To account for indication bias, an IPTW analysis was performed which again demonstrated lower mortality risk in statin versus nonstatin users. Among all statin users, we also observed differences in mortality risk based on individual statins and based on certain statin properties. The synthetic-derived statin users and the hydrophilic statin users had lower mortality than their respective comparison groups. Additionally, atorvastatin, pravastatin, and rosuvastatin users had lower mortality compared to simvastatin users.

Mortality from sepsis is high despite current treatment and management strategies. Improving sepsis survival and outcomes remains an important priority. Statins are widely prescribed given their indications and tolerability profile. Should a mortality benefit be found in patients taking a statin with sepsis, it would extend its benefits and suggest another potential indication in populations at higher risk for sepsis, such as patients with end stage kidney disease [10].

The pleotropic effects of statins have been well described, but a clear mechanism has not been established for statin benefit in sepsis [17]. Statins have been observed to demonstrate anti-inflammatory [18], antioxidative [19], immunomodulatory [20], and anticancer effects [21]. In addition, statins have been purported to have a direct antimicrobial effect [13, 22]. They have also been repurposed and explored as inhalation therapy for asthma in animal studies [23]. Furthermore, it has been observed that pretreatment with simvastatin (other statins were not investigating against simvastatin) improves sepsis survival in murine models by preservation of cardiac function, reduction of circulatory inflammatory cytokines, reduction of neutrophilic migration to the lungs, and improvement in T cell function [24–26]. With the many aforementioned effects that statins are theorized to exhibit, our findings further support a class-wide benefit of statins in sepsis patients. Potential benefits of statins have even been studied in COVID-19 patients, though statin use in this population has shown mixed results on whether they are associated with improved outcomes [27, 28].

In one of the largest retrospective studies to date, comparing statin users to nonusers in septic patients, Lee et al. found a similar mortality benefit among statin users [11]. It was also observed that simvastatin had the greatest mortality benefit when compared to atorvastatin and rosuvastatin, contrary to our study findings. Furthermore, in previously published studies, simvastatin was also found to have the most potent in vitro antimicrobial activity [13, 22]. In our study, we investigated and compared statins by biochemical property and observed that synthetic-derived and hydrophilic statins had lower mortality outcomes. Rosuvastatin was the only statin to belong to both the synthetic derivation and hydrophilic cohorts.

Randomized controlled trials investigating the association of statins and sepsis outcomes have been previously explored. In the randomized controlled trial (the SAILS study), authors investigated the use of rosuvastatin in patients with sepsis-associated acute respiratory distress syndrome (ARDS) but failed to demonstrate a mortality benefit [14]. This study population comprised of more critically ill patients with ARDS and differed from our population. Additionally, a meta-analysis of 5 randomized controlled trials (*n* = 650) performed by Pasin et al. failed to demonstrate mortality benefit in statin users when compared to nonusers [15]. The meta-analysis' study population was less critically ill than the SAILS study and categorized as either sepsis due to pneumonia, sepsis ward, or severe sepsis. Of the 5 studies analyzed, only atorvastatin and simvastatin were studied (4 investigated atorvastatin and 1 investigated simvastatin). This meta-analysis overall included a small number of patients compared to our study population of over 100,000 patients exposed to either of 5 different statins.

### 4.1. Limitations and Strengths

There are several potential limitations that may confound the interpretation of our findings. First, we did not have comprehensive information on medication adherence and used pharmacy dispensation information as a proxy. Furthermore, we did not have information on whether statin use was continued and thus whether patients were statin treated during hospitalization. This determination would have implications on a potential antibiotic effect of statins versus whether there is a conditioning benefit of statins during sepsis. We used primary hospitalization diagnosis codes to identify patients with sepsis and did not have microbiologic cultures or laboratory confirmation of source of infection. Our EHR-based approach did not use traditional clinical criteria to define sepsis [29]. We did not include specific organ infection codes when identifying and characterizing our study cohort. Our study cohort may have also included patients that were coded for sepsis, but had short stays for observation or surgical procedural stays where the proportion of statin users may have differed from the overall cohort. We also did not have the cause of death to determine whether mortality was related to sepsis for the population. Explicit ICD codes specific to sepsis were used to define our population. Thus, a limitation of this study is the inconsistency identifying sepsis throughout the study as its definition changed in the transition from ICD-9 to ICD-10. A sepsis population derived from ICD-9 coding may be different than those derived from ICD-10. In addition, we did not have data on the severity of sepsis on initial presentation such as a Sequential Organ Failure Assessment (SOFA) score or Acute Physiology and Chronic Health Evaluation (APACHE) score; thus, we could not determine the extent to which statin use mitigated the severity of sepsis or whether statin users overall were admitted with a less severe presentation of sepsis. Because SOFA scores vary in real time, we did not have the capability to access individual scores at or near the sepsis index date. We also did not factor ICU stay during the index hospitalization or receipt of mechanical ventilation into the outcome in our analyses. While we attempted to account for indication bias by employing an IPTW approach to control for confounding, this approach does not necessarily balance the populations on unobserved or unmeasured characteristics. Residual confounding also exists including heterogeneity of practice by different providers. We did not use categorial assignment of treatment facility as a proxy in the adjusted analyses.

Strengths of our study include the large, diverse population studied within a real-world setting that captured outcomes as part of routine clinical practice. The KPSC pharmacy analytic database is also another strength and includes prescription and refill data on 95% of members. Our statin use definition was rigorous requiring 2 documented pharmacy fills. Our data were collected from the KPSC electronic health records with a sepsis population exceeding over 100,000 patients. This study population was diverse comprised of Whites, Hispanics, Asians, and Blacks, supporting the generalizability of our findings. In addition, we performed a subgroup analysis among statin users only, thus minimizing a potential healthy user bias.

## 5. Conclusion

Among a large, diverse population of patients hospitalized for sepsis within a 10-year window, we observed that statin use was associated with lower mortality at 30 and 90 days. Additionally, among statin users, we found specific statins and statin classes were associated with better outcomes. Given the wide use and tolerability of statins, their use in high-risk populations may have extended benefits into infections and sepsis.

## Figures and Tables

**Table 1:** Pharmacological properties of statins.

Statin	Derivation	Property

Atorvastatin	Synthetic	Lipophilic
Fluvastatin	Synthetic	Lipophilic
Lovastatin	Fungal	Lipophilic
Pitavastatin	Synthetic	Lipophilic
Pravastatin	Fungal	Hydrophilic
Rosuvastatin	Synthetic	Hydrophilic
Simvastatin	Fungal	Lipophilic

**Table 2:** Population cohort characteristics. Baseline characteristics are included for the entire study population (those admitted to the hospital with sepsis). Comorbidities were determined as covariates as it relates to risk factors for sepsis mortality.

Baseline characteristics	Statin user (*n* = 34,088)	Nonstatin user (*n* = 94,073)	*P* value

Demographics			
Mean age (SD)	74.3 (11.4)	64.3 (19.0)	<0.001
Sex, male	18,386 (53.9%)	44,476 (47.3%)	<0.001
White	20,104 (59%)	45,278 (48.1%)	<0.001
Black	3,394 (10%)	11,699 (12.4%)	
Hispanic	7,338 (21.5%)	28,359 (30.1%)	
Asian	2,914 (8.5%)	7,234 (7.7%)	
Others	338 (1%)	1,503 (1.6%)	
Comorbidity			
Liver disease	2,904 (8.5%)	10,472 (11.1%)	<0.001
Chronic kidney disease	16,006 (47%)	24,040 (25.6%)	<0.001
Heart failure	9,568 (28.1%)	14,531 (15.4%)	<0.001
Chronic pulmonary disease	8,706 (25.5%)	19,219 (20.4%)	<0.001
Ischemic heart disease	14,246 (41.8%)	19,622 (20.9%)	<0.001
Rheumatological disease	867 (2.5%)	2,221 (2.4%)	0.06
Hypertension	31,236 (91.6%)	60,462 (64.3%)	<0.001
Cerebrovascular disease	4,688 (13.8%)	8,523 (9.1%)	<0.001
Diabetes mellitus	19,723 (57.9%)	33,074 (35.2%)	<0.001
Dementia	4,597 (13.5%)	10,162 (10.8%)	<0.001
Malnutrition	12,601 (37%)	31,745 (33.7%)	<0.001
History of transplantation	987 (2.9%)	2,006 (2.1%)	<0.001
History of GI bleed	5,452 (16%)	13,591 (14.4%)	<0.001
Alcohol/drug use	3,101 (9.1%)	11,528 (12.3%)	<0.001
AIDS/HIV	148 (0.4%)	573 (0.6%)	0.002
Hyperlipidemia	32,080 (94.1%)	50,931 (54.1%)	<0.001
Charlson comorbidity index			
0	2394 (7%)	25027 (26.6%)	<0.001
1	3927 (11.5%)	14383 (15.3%)	
2	5121 (15%)	13269 (14.1%)	
3	4450 (13.1%)	9646 (10.3%)	
≥4	18196 (53.4%)	31748 (33.7%)	
Medications			
Angiotensin receptor blockers	7,030 (20.6%)	9,177 (9.8%)	<0.001
Beta-blockers	19,475 (57.1%)	28,383 (30.2%)	<0.001
ACE inhibitors	17,256 (50.6%)	26,357 (28%)	<0.001
Systemic corticosteroids	8612 (25.3%)	21360 (22.7%)	<0.001
Disease modifying antirheumatic drugs	1,125 (3.3%)	3,101 (3.3%)	0.972

**Table 3:** Mortality risk comparison of statin use vs. nonuse of statins by biochemical property and of statins head-to-head.

HR (95% CI)	30-day mortality	90-day mortality

Statin use vs. nonuse	0.80 (0.77–0.83)	0.79 (0.77–0.81)
Statin properties		
Synthetic vs. fungal	0.86 (0.81–0.91)	0.85 (0.81–0.89)
Hydrophilic vs. lipophilic	0.90 (0.81–1.01)	0.86 (0.78–0.94)
Specific drugs		
Simvastatin (reference)		
Atorvastatin	0.87 (0.82–0.92)	0.85 (0.81–0.90)
Lovastatin	1.22 (1.10–1.36)	1.19 (1.08–1.31)
Pravastatin	0.87 (0.76–0.98)	0.83 (0.74–0.92)
Rosuvastatin	0.85 (0.66–1.10)	0.77 (0.62–0.97)

Multivariable regressions modeling used adjusting for age, gender, race, liver disease, chronic kidney disease, heart failure, ischemic heart disease, chronic pulmonary disease, rheumatological disease, hypertension, cerebrovascular disease, diabetes mellitus I and II, dementia, malnutrition, peptic ulcer disease, all malignancy, organ transplantation, history of gastrointestinal hemorrhage, alcohol/drug use, HIV/AIDS, hyperlipidemia, biologic use, corticosteroid use, and disease modifying antirheumatologic drug use.

## Data Availability

The data analyzed during this study are included within the article.

## Additional Points

Statin users compared to nonstatin users were associated with 20% lower 30-day mortality in patients hospitalized with sepsis. Synthetic derived and hydrophilic statins were associated with better outcomes.

## Supplementary Materials

Supplementary Materials Supplementary Table 1: diagnosis codes used to identify sepsis patients and also comorbid conditions. Supplementary Table 2: generic medication names used to extract medication information. Supplementary Table 3: clinical outcomes by different statin properties and derivation.
