# Supplementary material for: Statin Use and Mortality among Patients Hospitalized with Sepsis: A Retrospective Cohort Study within Southern California, 2008–2018
Source: Crit Care Res Pract. 2022 May 6;2022:7127531. doi: 10.1155/2022/7127531 (PMC9106495; doi:10.1155/2022/7127531)
Supplement: Supplementary Materials — Supplementary Table 1: diagnosis codes used to identify sepsis patients and also comorbid conditions. Supplementary Table 2: generic medication names used to extract medication information. Supplementary Table 3: clinical outcomes by different statin properties and derivation. [file 7127531.f1.docx]

**Supplemental Table 1. Diagnosis Codes for Data Abstraction**

| **Variable** | **CPT or ICD-9 / ICD-10 codes** |
| --- | --- |
| Sepsis | ICD 9: 670.20, 670.22, 670.24, 995.91, 995.92  ICD 10: A02.1, A22.7, A26.7, A32.7, A40.0, A40.1, A40.3, A40.8, A40.9, A41.01, A41.02,  A41.1, A41.2, A41.3, A41.4, A41.50, A41.51, A41.52, A41.53, A41.59, A41.81, A41.89,  A41.9, A42.7, A54.86, B37.7, O03.37, O03.87, O04.87, O07.37, O08.82, O85, O86.04,  P36.0, P36.10, P36.19, P36.2, P36.30, P36.39, P36.4, P36.5, P36.8, P36.9, R65.20,  R65.21, T81.44XA, T81.44XD, T81.44XS |
| Liver disease | ICD9 571.8, 571.9, 572.8  ICD10 K70.9, K71.0, K71.10, K71.11, K71.2, K71.3, K71.4, K71.5, K71.51, K71.6, K71.7, K71.8, K71.9, K75.89, K75.9, K76.9, P78.84 |
| Chronic Kidney Disease | ICD9 585.6, 642.11, 642.12, 642.13, 642.14, 646.20, 646.21, 646.22, 646.23  ICD 10 N18.6, O26.831, O26.832, O26.833, O26.839 |
| Pulmonary Disease | ICD9 031.0  ICD10 J44.1, J44.9, J84.89, J84.9 |
| Rheumatologic Disease | ICD9 714.0, 714.32, 714.33, 714.81, V82.1  ICD10 M05.10, M05.112, M05.119, M05.121, M05.122, M05.129, M05.131, M05.132, M05.139, M05.141, M05.142, M05.149, M05.151, M05.152, M05.159, M05.161, M05.162, M05.169, M05.171, M05.172, M05.179, M05.19, M05.20, M05.211, M05.212, M05.219, M05.221, M05.222, M05.229, M05.231, M05.232, M05.239, M05.241, M05.242, M05.249, M05.251, M05.259, M05.261, M05.262, M05.269, M05.271, M05.272, M05.279, M05.29, M05.30, M05.311, M05.312, M05.319, M05.321, M05.322, M05.329, M05.331, M05.332, M05.339, M05.341, M05.342, M05.349, M05.351, M05.352, M05.359, M05.361, M05.362, M05.369, M05.371, M05.372, M05.379, M05.39, M05.40M05.411 , M05.412, M05.419, M05.421, M05.422, M05.429, M05.431, M05.432, M05.439, M05.441, M05.442, M05.449, M05.451, M05.452, M05.459, M05.461, M05.462, M05.469, M05.471, M05.472, M05.479, M05.49, M05.50, M05.511, M05.512, M05.519, M05.521, M05.522, M05.529, M05.531, M05.532, M05.539, M05.541, M05.542, M05.549, M05.551, M05.552, M05.559, M05.561, M05.562, M05.569, M05.571, M05.572, M05.579, M05.59, M05.80, M05.811, M05.812, M05.819, M05.821, M05.822, M05.829, M05.831, M05.832, M05.839, M05.841, M05.842, M05.849, M05.851, M05.852, M05.859, M05.861, M05.862, M05.869, M05.71, M05.872, M05.879, M05.89, M05.9, M06.00, M06.011, M06.012, M06.019, M06.021, M06.022, M06.029, M06.031, M06.032, M06.039, M06.041, M06.042, M06.049, M06.051, M06.052, M06.059, M06.062, M06.069, M06.071, M06.072, M06.079, M06.08, M06.09, M06.20, M06.211, M06.212, M06.219, M06.222, M06.229, M06.231, M06.232, M06.239, M06.241, M06.242, M06.249, M06.251, M06.252, M06.259, M06.261, M06.262, M06.269, M06.271, M06.272, M06.279, M06.28, M06.29, M06.30, M06.311, M06.312, M06.319, M06.321, M06.322, M06.329, M06.331, M06.332, M06.339, M06.341, M06.342, M06.349, M06.351, M06.352, M06.359, M06.361, M06.362, M06.369, M06.371, M06.372, M06.379, M06.38, M06.39, M06.80, M06.811, M06.812, M06.819, M06.821, M06.822, M06.829, M06.831, M06.832, M06.839, M06.841, M06.842, M06.849, M06.851, M06.852, M06.859, M06.861, M06.862, M06.869, M06.871, M06.872, M06.879, M06.88, M06.89, M06.9, M06.00, M08.00, M08.011, M08.012, M08.019, M08.021, M08.022, M08.029, M08.031,M08.032,M08.039, M08.041, M08.042, M08.049, M08.051, M08.052, M08.059, M08.061, M08.062, M08.069, M08.071, M08.072, M08.079, M08.08, M08.09, M08.20, M08.211, M08.212, M08.221, M08.222, M08.229, M08.231, M08.232, M08.239, M08.241, M08.242, M08.249, M08.251, M08.252, M08.252, M08.259, M08.261, M08.262, M08.269, M08.271, M08.272, M08.279, M08.28, M08.3, M08.40, M08.411, M08.412, M08.419, M08.421, M08.422, M08.429, M08.431, M08.432, M08.439, M08.441, M08.442, M08.449, M08.451, M08.452, M08.459, M08.61, M08.462, M08.469, M08.471, M08.472, M08.479, M08.48 |
| Ischemic Heart Disease | ICD9 411.89, 414.8, 414.9, V17.3, V81.0  ICD10 I24.8, I24.9, I25.89, I25.9 |
| Angina | ICD9 413.0, 413.1, 413.9  ICD10 I20.0, I20.1, I20.8, I20.9, I23.7, I25.700, I25.701, I25.708, I25.709, I25.710, I25.718, I25.719, I25.720, I25.728, I25.729, I25.738, I25.739, I25.750, I25.760, I25.790, I25.791, I25.798, I25.799, I25.810 |
| Myocardial Infarction | ICD9 411.0, 412, 429.79  ICD 10 I21.3, I21.4, I21.9, I21.A1, I21.A9, I22.2, I25.2 |
| Percutaneous coronary/coronary artery bypass graft intervention | ICD 9 996.03, V45.81  ICD 10 Z95.1 |
| Heart Failure | ICD9 398.91, 402.01, 402.11, 428.0, 428.1, 428.20, 428.21, 428.22, 428.23, 428.30, 428.31, 428.32, 428.33, 428.40, 428.41, 428.42, 428.9  ICD10 I09.81, I11.0, I11.9, I50.20, I50.21, I50.22, I50.23, I50.30, I50.31, I50.32, I50.33, I50.811, I50.812, I50.813, I50.814, I50.82, I50.83, I50.84, I50.89, I50.9, I97.130, I97.131 |
| Cerebrovascular Disease/Stroke | ICD9 436, 437.1, 438.8, 437.9, 438.0, 438.11, 438.12, 438.81, 438.82, 438.84, 438.85, 438.89, 674.03, 674.04  ICD10 G46.8, I67.858, I67.89, I67.9, I68.8, I69.80, I69.81, I69.811, I69.820, I69.822, I69.823, I69.890, I69.891, I69.892, I69.893, I69.898, I69.90, I69.91, I69.920, I69.921, I69.922, I69.923, I69.990, I69.991, I69.992, I69.993, I69.998 |
| Cerebral atherosclerosis | ICD 9 437.0  ICD 10 I67.2 |
| Bedridden Status | ICD9: V49.84  ICD10: Z74.01 |
| Obesity | ICD9 278.00, 278.01, 278.03, 649.11, 649.12, V77.8  ICD10 E66.01, E66.09, E66.1, E66.2, E66.8, E66.9, O99.210, O99.211, O99.212, O99.213, O99.214, O99.215 |
| Diabetes | ICD 9: 250.0  ICD 10: E10.9, E11.9 |
| Dementia | ICD 9 290.0, 290.10, 290.11, 290.12, 290.12, 290.13, 290.20, 290.21, 290.3, 290.40, 290.41, 290.42, 290.43, 291.2, 292.82, 294.1, 294.20, 294.21, 331.19, 331.82  ICD 10 F01.50, F01.51, F02.80, F02.81, F02.90, F03.91, F10.27, F10.97, F13.27, F13.97, F18.17, F18.27, F18.97, F19.17, F19.27, F19.97, G31.09, G31.83 |
| Hemiplegia or paraplegia | ICD 9 334.1, 342.00, 342.01, 342.02, 342.10, 342.11, 342.12, 342.80, 342.81, 342.82, 342.90, 342.91, 342.92, 343.4, 344.00, 344.01, 344.02, 344.03, 344.09, 344.1, 344.2, 344.30, 344.31, 344.32, 344.40, 344.41, 344.42, 344.5, 367.52, 376.22, 378.55, 378.56, 378.72, 378.86, 780.72  ICD 10 G04.1, G11.4, G23.1, G81.00, G81.01, G81.02, G81.03, G81.04, G81.10, G81.11, G81.12, G81.13, G81.14, G81.90, G81.91, G81.92, G81.93, G81.94, G82.20, G82.21, G82.22, G82.50, G82.51, G82.52, G82.53, G82.54, G83.0, G83.10, G83.11, G83.12, G83.13, G83.14, G83.20, G83.21, G83.22, G83.23, G83.24, G83.30, G83.31, G83.32, G83.33, G83.34, H49.30, H49.31, H49.32, H49.33, H49.40, H49.41, H49.42, H49.43, H51.20, H51.21, H51.22, H51.23, H52.511, H52.512, H52.513, H52.519, R53.2 |
| Malnutrition | ICD 9: 263.9  ICD 10: E46 |
| Peptic ulcer disease | ICD 9 V12.71  ICD 10 Z87.11 |
| Any malignancy including leukemia or lymphoma | ICD 9: 208.9, 208.0, 208.8, 205.8, 205.1, 205.0, 208.1, 207.8, 207.2, 205.9, 205.2, 202.4, 206.9, 204.9, 204.0, 204.8, 204.1, 204.2, 204.0, 204.9, 204.0, 207.8, 207.2, 206.0, 206.9, 206.8, 206.0, 206.1, 206.2, 205.0, 205.9, 205.8, 205.2, 203.1, 208.2, 208.0, 202.0, 202.8, 200.2, 785.6, 201.9, 200.8, 200.7, 200.6, 200.0, 200.1, 200.4, 200.3, 200.5, 202.7, 202.1, 202.0  ICD 10: C95.0, C95.00, C95.01, C95.02, C95.10, C95.11, C95.12, C95.90, C95.91, C95.92, C85.10, C85.11, C85.12, C85.13, C85.14, C85.15, C85.16, C85.17, C85.18, C85.19, C85.20, C85.21, C85.22, C85.23, C85.24, C85.25, C85.26, C85.27, C85.28, C85.29, C85.80, C85.81, C85.82, C85.83, C85.84, C85.85, C85.86, C85.87, C85.88, C85.89, C85.90, C85.91, C85.92, C85.93, C85.94, C85.95, C85.96, C85.97, C85.98, C85.99, C91.5, C84.7, C84.6, C86.6, C86.5, C85.1, C83.5, C88.34, C86.4, C83.7, C83.1, C82.6, C84.A, C82.5, C83.3, C86.2, C88.4, C86.0, C82.9, C82.0, C82.1, C82.2, C82.3, C82.4, C82.8, C86.1, C85.9, C96.A, C80.1 C81.9, C81.3, C81.4, C81.2, C81.0, C81.1, C81.7, C83.8, C84.4, C83.5, C84.4, C83.0, C88.0, C88.4, C83.1, C84.9, C84.Z, C85.2, C88.3, C88.4, C84.9, C83.0, C83.9, C83.8, C85.9, C85.8, C83.0, C84.4, C86.6, C83.8, C88.4, C83.0, C86.3, C83.5, C96.A |
| Peripheral arterial disease | ICD 9: 443.9  ICD 10: I73.9 |
| Solid organ transplantation such as heart or renal | ICD 9: V42.0, V42.1, V42.6, V42.7  ICD 10: Z94.0, Z94.1, Z94.2, Z94.3, Z94.4, Z94.83 |
| Gastrointestinal or esophageal hemorrhage | ICD 9: 578.9, 459.0, 578.0, 578.1, 578.9, 537.84, 530.82, 569.86, 537.84, 456.0  ICD 10: K92.2, K29.01, K62.5, K31.811, I85.01, K22.8 |
| Alcohol/drug use | ICD 9 303.92, 303.93, 305.00 , 305.01, 305.02, 305.03, 357.5, 425.5, 535.30, 535.31  ICD 10 F10.10, F10.11, F10.120, F10.121, F10.129, F10.14, F10.150, F10.151, F10.159, F10.180, F10.181, F10.182, F10.188, F10.19, F10.20, F10.21, F10.220, F10.221, F10.229, F10.230, F10.231, F10.232, F10.239, F10.24, F10.250, F10.251, F10.259, F10.26, F10.27, F10.280, F10.281, F10.282, F10.288, F10.29, F10.920, F10.921, F10.929, F10.94, F10.950, F10.951, F10.959, F10.96, F10.97, F10.980, F10.981, F10.982, F10.988, F10.99 |
| AIDS/HIV | ICD 9 042, 079.53, 795.71, V08  ICD 10 B20, B97.35, Z71.7, Z83.0 |

**Supplemental Table 2. Drug Classes and Generic Drug names for Data Abstraction**

| **Drug Class** | **Generic drug names** |
| --- | --- |
| Angiotensin II antagonists | Losartan, valsartan, irbesartan, candesartan, telmisartan, eprosartan, olmesartan |
| Calcium channel blocker | Nicardipine, diltiazem, nimodipine, verapamil, nidefipine, isradipine, amlodipine, nisoldipine, clevidipine |
| Systemic immunosuppressive agents | Prednisone, budesonide, prednisolone, tofacitinib, cyclosporine, tacrolimus, sirolimus, everolimus, azathioprine, leflunomide, mycophenolate, abatacept, adalimumab, anakinra, certolizumab, etanercept, golimumab, infliximab, ixekizumab, natalizumab, rituximab, secukinumab, tocilizumab, ustekinumab, vedolizumab, basiliximab, daclizumab |
| Beta-blockers | Acebutolol, atenolol, betaxolol, bisoprolol, carteolol, esmolol, metoprolol, penbutolol, nadolol, nebivolol, pindolol, propranolol, timolol, sotalol, carvedilol, labetalol |
| Angiotensin converting enzyme inhibitors | Fosinopril, captopril, moexipril, lisinopril, ramipril, quinapril, enalapril, benazepril, trandolapril, perindopril, |
| Loop diuretics | Bumetanide, ethacrynic acid, torsemide, furosemide |
| Disease-modifying antirheumatic drugs (DMARD) | Abatacept, adalimumab, anakinra, azathioprine, chloroquine, ciclosporin, d-penicillamine, etanercept, golimumab, hydroxychloroquine, infliximab, leflunomide, methotrexate, minocycline, rituximab, sulfazalasine, tocilizumab, tofacitinib |
| Systemic corticosteroids | Prednisone, prednisolone, methylprednisolone, beclomethasone, dexamethasone |
| Proton pump inhibitors | Dexlansoprazole, esomeprazole, pantoprazole, rabeprazole, lansoprazole, omeprazole |
| Antipsychotics | Thioridazine, pimozide, molindone, haloperidol, fluphenazine, risperidone, olanzapine, aripiprazole, paliperidone, clozapine, asenapine, olanzapine |

**Supplemental Table 3:** Outcomes by statin property and class

| **Type of Statin** | **N** | **No. died at 30 days** | **No. died at 90 days** | **Length of stay** |
| --- | --- | --- | --- | --- |
| Fungal derived | 20,802 | 3,220 (15.5%) | 4,447 (21.4%) | 6.8 SD (9.3) |
| Synthetic derived | 13,286 | 1,812 (13.6%) | 2,504 (18.8%) | 6.3 SD (8.2) |
| Lipophilic | 31,735 | 4,699 (14.8%) | 6,507 (20.5%) | 6.6 SD (9.0) |
| Hydrophilic | 2,353 | 333 (14.2%) | 444 (18.9%) | 6.8 SD (7.4) |
| Simvastatin | 16,888 | 2,574 (15.2%) | 3581 (21.2%) | 6.8 SD (9.7) |
| Atorvastatin | 12,863 | 1753 (13.6%) | 2425 (18.9%) | 6.3 SD (8.1) |
| Lovastatin | 1,975 | 372 (18.8%) | 498 (25.2%) | 6.5 SD (7.8) |
| Pravastatin | 1,939 | 274 (14.1%) | 368 (19%) | 6.7 SD (7.1) |
| Rosuvastatin | 414 | 59 (14.3%) | 76 (18.4%) | 7.2 SD (8.8) |

Table of outcomes by statin includes number of patients who died at 30 days, number of patients who died at 90 days, and length of stay. SD= standard deviation.
